# Host Antiviral Responses against Avian Infectious Bronchitis Virus (IBV): Focus on Innate Immunity

**DOI:** 10.3390/v13091698

**Published:** 2021-08-26

**Authors:** Yun Zhang, Zhichao Xu, Yongchang Cao

**Affiliations:** State Key Laboratory of Biocontrol, School of Life Sciences, Sun Yat-sen University, Guangzhou 510006, China; zhangyun6@mail.sysu.edu.cn (Y.Z.); xuzhich5@mail.sysu.edu.cn (Z.X.)

**Keywords:** innate immunity, avian infectious bronchitis virus, pattern recognition receptor, interferons, chicken

## Abstract

Avian infectious bronchitis virus (IBV) is an important gammacoronavirus. The virus is highly contagious, can infect chickens of all ages, and causes considerable economic losses in the poultry industry worldwide. In the last few decades, numerous studies have been published regarding pathogenicity, vaccination, and host immunity-virus interaction. In particular, innate immunity serves as the first line of defense against invasive pathogens and plays an important role in the pathogenetic process of IBV infection. This review focuses on fundamental aspects of host innate immune responses after IBV infection, including identification of conserved viral structures and different components of host with antiviral activity, which could provide useful information for novel vaccine development, vaccination strategies, and intervention programs.

## 1. Introduction

The avian infectious bronchitis virus (IBV) belongs to the genus *Gammacoronavirus*. This virus infects the upper respiratory tract, reproductive system, and kidneys of chickens [1]. Depending on the strain, IBV can also be found in the epithelium of the urogenital tract [2], intestinal tract [3], enterocytes of ileum and rectum [4], and glandular epithelium of proventriculus [5]. Chickens of all ages are susceptible to the virus, while young chickens presented more severe clinical signs compared to older ones [6]. After IBV infection, subsequent bacterial infections often occur, thus resulting in high mortality [7,8]. Therefore, IBV infection is considered to be the second most damaging poultry disease worldwide [9].

The IBV viral genome is an unsegmented, single-stranded positive-sense RNA of 27.6 kb in length [10]. The 3′-end of the viral genome encodes structural and accessory proteins in a sequence of spike (S), accessory proteins 3a, 3b, envelope (E), membrane (M), accessory proteins 4b, 5a, 5b, and nucleocapsid (N). The 5′-end, which encompasses approximately two-thirds of the viral genome, encodes two overlapping replicase proteins (polyprotein 1a and 1b) that are further processed into 15 non-structural proteins (Nsp2–Nsp16) [11]. 

As for control of IBV infection, strict biosecurity on poultry farms is required. Because no effective drugs against IBV infection have been reported so far, vaccination is the most efficient approach to prevent production losses [12]. However, due to error-prone viral genome replication and poor cross-protection among IBV strains of different serotypes, current vaccines are inadequate to offer full protection [12]. Furthermore, the importance of chicken breed in IBV resistance was also discussed [2], and the MHC B locus was known to play a crucial role in susceptibility to the virus [13]. Detrimental local inflammatory responses in certain chicken breeds, for instance, 335/B19, might be associated with susceptibility to IBV [14]. Despite differential innate immune responses after IBV infection in different chicken lines, viral loads were similar in the tracheas of these chicken lines [15].

Due to the requirement for reducing and limiting antibiotics in the poultry industry, IBV control is of great importance in terms of the increasing demand for antibiotic-free chickens. Because unsuccessful vaccination will lead to failure in IBV control, in the last few decades, numerous studies have presented the function of host innate immunity in resistance towards IBV. The innate immune system, containing various cells and molecules, non-specifically targets invading pathogens and serves as the first line of defense. Therefore, besides the efforts in novel vaccine development and prophylactic measures, the enhancement of innate immune responses in resistance to IBV have gained increasing attention in the design of novel prevention approaches.

## 2. Cells Involved in Innate Immunity after IBV Infection

Although IBV was the first coronavirus discovered, its functional host cell receptor is still in debate. Previous research suggests that Neu5Acα2-3Galβ1-3GlcNAc (Neu5Ac) (one sialic acid containing glycan) could be a potential receptor, as it bound specifically to the S1 protein of the M41 IBV strain [16]. Aminopeptidase N (APN), also known as CD13, is also suggested to be a possible functional receptor, though results of the IBV entry experiments with chicken APN transfection were quite contradictory [16]. Two high-affinity peptides, H (HDYLYYTFTGNP) and T (TKFSPPSFWYLH) of APN, were shown to bind IBV S1 antibodies and reduced IBV proliferation in chicken embryo kidney (CEK) cells and chicken tracheas, lungs, and kidneys, presenting an alternative approach to IB prevention or treatment [17]. In addition, membrane protein heat shock protein member 8 (HSPA8) [18] and clathrin-mediated endocytosis [19] played an important role in IBV attachment and entry.

IBV primarily targets epithelial cells, causing mucosal pathological lesions, including epithelial hyperplasia, ciliary loss and necrosis, and inflammatory cell infiltration [16]. After viral entry, hyperplasia of goblet cells and alveolar mucous glands also occurs, while depletion of goblet cells and alveolar mucus glands were observed at 5 day-post-infection (dpi) in specific pathogen-free (SPF) chickens [20]. Later, phagocytic heterophils and macrophages are recruited.

Heterophils, counterpart to mammalian neutrophils, are main granulocytes in poultry blood and form the polymorphonuclear cell population of chicken [21,22]. In SPF chickens, within 24–72 h-post-infection (hpi), numerous heterophils were recruited to IBV infection sites [23]. Granule contents including cathepsin S and heterophil extracellular traps (HETs) are released by heterophil degranulation, resulting in pathogen destruction and neutralization in blood [24]. The heterophils-depleted chickens suffered from more severe nasal exudation during IBV infection [6], indicating that heterophils play an important role in the resistance to IBV infection.

Macrophages are involved in recognition, phagocytosis, and degradation of invading pathogens. The number of macrophages in respiratory lavage fluid, embryonic tissues, trachea, and lung was increased after IBV inoculation [25,26]. In respiratory tracts, the increased macrophages were accompanied by reduced IBV viral loads, as well as production of interleukin-1β (IL-1β) [27]. In vitro studies showed a decreased viability and phagocytic ability of HD11 chicken macrophage cells and chicken peripheral blood mononuclear cell-derived macrophages (PBMCs-Mφ) after infection by the respiratory M41 IBV strain. Several genes including IFN-α, IL-1β, IL-6, etc. were significantly increased in these IBV-infected macrophages, and apoptosis was triggered by virus replication [28]. Overall, these results suggest macrophages play an important role in host innate and acquired immunity to resist IBV infection. In addition, expression profiling of mRNAs and long non-coding RNAs (lncRNAs) in Beaudette IBV strain-infected HD11 cells indicated that the differentially expressed (DE) mRNAs and DE lncRNAs were mainly involved in innate immunity and amino acid/nucleic acid metabolism [29]. The competing endogenous RNA (ceRNA) network based on two differentially expressed miRNAs, namely gga-miR-30d and miR-146a-5p [30], was established [29]. Gga-miR-30d was demonstrated to inhibit IBV replication in the HD11 cells by targeting USP47 [31], whereas miR-146a-5p promoted IBV replication in the HD11 cells through IRAK2 and TNFRSF18 [32]. These results further provide valuable information for new therapeutic approaches to IBV control. However, not all IBV strains can infect HD11 effectively. Moreover, the IBV strain used mostly in in vitro studies was cell-adapted Beaudette IBV strain, which is not pathogenic in chickens. Therefore, other cell lines (e.g., MQ-NCSU macrophage cells [33]), macrophages isolated from chickens of different ages, IBV strains that propagate well in both macrophages and chicken, and novel techniques (e.g., single cell sequencing [34]) are needed to fully elucidate IBV pathogenesis. 

Other innate immune cells, such as natural killer (NK) cells, are also activated at an early IBV infection stage. For instance, 5-week-old chickens infected with M41 IBV strain via nasal–tracheal route induced a rapid activation of CD107^+^CD3^−^ NK cells in the lungs and blood from 1 dpi [35]. These cells are best known for their abilities in cytokine production and cytotoxicity, which are important for innate defense against viral infections. In contrast to mammalian NK cells, the frequency of avian NK cells in peripheral epithelial lymphocytes (PBL) and spleen was low (0.5–1.0%) [36]. Therefore, future work is required to understand the relationship between avian NK cells and IBV resistance.

To summarize, innate immune cells including heterophils, macrophages, and NK cells are recruited rapidly (24 hpi) at the sites of IBV infection. Based on the antiviral function of these cells, including pathogen recognition, phagocytosis, cytokine production, apoptosis regulation, etc., rapid activation of these cells is important for virus clearance and resistance to IBV.

## 3. Pattern Recognition Receptors Triggered by IBV Infection

### 3.1. PRRs in Chicken

Though less studied, chicken innate immunity functions are similar to mammalian innate immunity with certain differences. Pattern recognition receptors (PRRs) are important components in host innate immunity, which are expressed on cell membranes, endosome membranes, and lysosome membranes, as well as in cytoplast. Based on the mammalian literature, toll-like receptors (TLRs), retinoic acid-inducible gene I (RIG-I)-like receptors (RLRs), and NOD-like receptors (NLRs) are the most common host PRRs responding to viruses [37,38]. 

All identified TLRs are type I transmembrane proteins, enriched on the cell surface or membrane of endosomes [39]. In chickens, the currently known TLRs are chTLR-1A, chTLR-1B, chTLR-2A, chTLR-2B, chTLR-3, chTLR-4, chTLR-5, chTLR-7, chTLR-15, and chTLR-21 [40]. chTLR15 [41] and chTLR 21 [42] are unique TLRs in chicken. In laying hens, interaction of chTLRs and their specific ligands leads to expression and secretion of pro-inflammatory cytokines and avian β-defensins (AvBDs) [43]. 

In mammals, TLR3 and TLR7 are well known for recognition of RNA viruses [44]. chTLR3 and chTLR7 are orthologous to their mammalian counterparts [45,46] and function similarly to stimulate production of type I interferons (IFNs) [16], though orthologs of mammalian IKKγ, MKK7, and IRF3 were not detected in chicken [45]. The significance of chTLRs in immune responses is summarized and the review is recommended for further reading [47].

The mammalian RLR family has three members: RIG-I, melanoma differentiation-associated gene 5 (MDA5), and laboratory of genetics and physiology 2 (LGP2) [48]. However, RIG-I is absent from the chicken genome [49]. MDA5 and LGP2 have been identified in chicken [50], which are involved in sensing dsRNA in chicken cells [51]. 

NLRs could also be additional candidate receptors in chicken because NLR elements (NALP1, NOD1, etc.) are identified in the chicken genome [52]. Expression and function of nucleotide-binding oligomerization domain-containing protein-1 (NOD1) in chicken were recently analyzed [53]. Different avian PRRs for virus recognition are summarized and the review is recommended for further reading [54].

### 3.2. IBV Infection Triggered PPR Activation

Lack of available cell lines of IBV strains has limited investigation of avian PRRs sensing the virus, and cell-adapted Beaudette IBV strains are extensively used in the research. In vivo studies using CEK cells and tracheal organ culture (TOC) presented elevated levels of chTLR3 and chMDA5 expression at an early stage of infection (9 hpi) by different respiratory or nephropathogenic IBV strains [55,56], while chTLR7 signaling was not affected in CEK cells [55]. Later (>18 dpi), it was shown that the respiratory M41 IBV strain could delay detection of MDA5 in CEK cells [56], and its ability to cleave MAVS allows the virus to escape from chMDA5–mediated antiviral responses [57]. Because RIG-I is absent in chickens, it is worthwhile to investigate the effect of nephropathogenic IBV strains in chMDA5 singling, which would provide useful information for control of the virus. 

In general, in vivo experiments presented activated PRR (mostly on chTLRs) expression after infection. For instance, in tracheas of 3-week-old and 6-day-old SPF chickens, up–regulation of chTLR3 and chTLR7 was observed at 1 dpi, when inoculated with respiratory attenuated Mass and Conn IBV strains, respectively [16], while down–regulation of chTLR3 was observed at 12 hpi in Conn IBV strain infection [26]. Activation of chTLR-1 LA, chTLR-1 LB, chTLR2, and chTLR6 was also observed from 1 dpi [58,59]. However, after inoculation with respiratory Brazilian field isolated IBV strain was inoculated, a suppressive effect on the TLR7 pathway was found at 1 dpi in 28-day-old chickens, while TLR3 was upregulated from 1 dpi. In this study, expression of TLR7 in trachea rose from 5 dpi [60]. At 3 dpi, expression of chTLR3 was induced, whereas chTLR7 was not affected, after inoculation with virulent M41 IBV strain [61]. At 9–10 dpi, in the splenic immune system of laying chickens, the expression level of TLR7 was significantly downregulated after infection with a respiratory T IBV strain at 9–10 dpi [62]. Nephropathogenic IBV strains were used to explore PRRs expression in kidneys. At 5 dpi, transcriptome analysis showed upregulation of MDA5 in 15-day-old SPF chickens [63]. Inoculation with two other Chinese field isolated nephropathogenic IBV strains (AH and TM) inhibited activation of the TLR7-MyD88 pathway, but not the TLR3-TIRF pathway at 5 dpi [64]. So far, there are few studies regarding kidney damage. Future work is required to fully elucidate the pathogenesis of the nephropathogenic IBV strains. Overall, considering the differences in PRR expression, virulence of IBV strains should be taken into consideration in field control of the virus.

Though these studies presented spatial and temporal differences in PRR regulation after IBV infection, due to the importance of PRRs in pathogen recognition, activation of PRRs is still important in IBV control. Therefore, to rapidly activate PRRs, TLR ligands have drawn attention in novel vaccine design. For instance, administration of TLR7 agonist R848 (resiquimod) with live or inactivated Mass IBV strain significantly enhanced the sIgA responses through upregulation of TGF-β4 [65]. In addition, although TLR21 recognizes microbial DNA containing unmethylated cytosine-guanosine deoxynucleotide (CpG) motifs [42], in ovo delivery of CpG oligodeoxynucleotides (ODNs) impaired IBV Ark99 (respiratory) replication in 18-day-old chicken embryos from 24 hpi [25]. Furthermore, in HD11 cells, stimulation with CpG ODNs triggered chicken TLR-21, which further induced NF-κB signaling pathway [42]. These findings may contribute to novel in ovo vaccine designs for neonatal chickens, and application of other TLR ligands requires future work. 

## 4. Cytokine Responses Triggered by IBV Infection

In general, PRR activation mediates IFN pathways via TRIF or MyD88. Subsequently, these signaling cascades promote the NF-κB and IFN regulatory factor (IRF) family, resulting in expression of type I IFNs and then transcription of interferon-stimulated genes (ISGs) with antiviral activity [66]. ISGs have diverse functions, including resisting and controlling pathogens, which form a complex web of host defenses [67].

In chickens, eight members of the IRF family were identified, whereas IRF3 and IRF9 are absent [68]. chIRF7 and chIRF1 are involved in chicken antiviral innate immunity [69,70] and chIRF1 functions as a critical IFN-β regulator against viral infection [70]. 

### 4.1. IBV Infection Triggered Interferon Activation

IFNs, including type I, type II, and type III IFNs [71], are multifunctional in the innate immune system. In general, type I IFNs (IFN-α, IFN-β, etc.) and type III IFNs (IFN-λ) have been proven for antiviral activity, while type II IFNs (IFN-γ) could activate T cells and macrophages [72].

The type I IFN functions as a powerful antiviral mechanism, which has been shown to be involved in host response after IBV infection. It was shown that IFN-α could inhibit respiratory Beaudette or Gray IBV strains both in vitro and in vivo [73]. In vitro studies showed that the induction of IFN-β is in an MDA5-dependent manner [56]. At an early infection stage (9 hpi), IFN-β was upregulated when infected with a nephropathogenic IBV strain [55]. However, when respiratory M41 IBV strain was used, the expression of IFN-β in CEK cells was delayed until 12 dpi, while accessory protein 5b was involved in induction of host shutoff that resulted in reduction of IFNs [74]. Furthermore, respiratory Beaudette IBV strain was shown to interfere with IFN-β-induced translocation of STAT1 and STAT1 phosphorylation in Vero cells at late stages of infection (18 hpi), ref. [74] suggesting respiratory IBV-mediated inhibition of IFN signaling in a time-dependent manner. The differences in IFN expression between respiratory and nephropathogenic IBV infections requires future work, which may help to understand the mechanisms underlying the tissue tropism of different IBV strains.

In vivo studies showed a more complex result, in that the expression level of IFN-α was significantly upregulated at 1 dpi in spleens after virulent respiratory IBV infection [62], while in tracheas the upregulation of IFNs was not observed at 3 dpi [61]. Furthermore, chickens vaccinated with attenuated respiratory M41 or LDT3 posed stronger type I IFN levels, respectively [61]. The virulence of IBVs might be the reason for the differences in IFN levels. Consistent with the results in PRR expression, these results also suggest it is important that the virulence of IBV strains be taken into consideration in field control of the virus.

Similarly to type I IFNs, at an early stage of infection (12 hpi), after inoculation with the respiratory Conn IBV strain, IFN-γ was significantly downregulated in tracheas and lungs of the infected chicken [26]. At 2–3 dpi, when inoculated with respiratory M41 IBV strain, IFN-γ was induced in tracheas and lungs [35,75]. Though the antiviral activity of IFN-γ against IBV has not been fully characterized, based on results observed in avian influenza virus (AIV) infected chickens, it was suggested that IFN-γ might indirectly interfere with IBV replication through the initiation of ISG-encoded ribonuclease L (RNase L) [76]. For expression of ISGs, studies showed that in different systems including chicken embryos (6 hpi), tracheas (3 dpi), and kidneys (5–6 dpi), upregulation of ISGs was presented in transcriptional analysis after infection with different respiratory IBV strains [61]. 

To summarize, though the responses of IFNs after IBV infection vary in a strain-dependent and time-dependent manner, in general, activation of IFNs is restrained at a very early stage of IBV infection to allow viral replication. Upregulation of IFNs is often observed with activated ISGs after infection is established, when the innate immunity responds for viral clearance. Therefore, early intervention and activation of IFNs are critical in control of the disease. 

### 4.2. IBV Infection Triggered Other Cytokine and Chemokine Activation 

Other cytokines and chemokines are also crucial regulators of innate immune responses against viral infection. For instance, correlated with recruited macrophages, production of IL-1β was involved in reducing IBV viral loads in the respiratory tract [27]. In addition, upregulation of IFN-α, IFN-γ, and IL12 at 12 hpi, upregulation of IFN-γ, IL-8, and macrophage inflammatory protein (MIP)-1β at 48 hpi, and upregulation of IFN-γ and IL-6 at 72 hpi were also observed, and the upregulation of these cytokines was associated with inhibition of respiratory IBV Ark99 replication [77].

Depending on the IBV strain, it was reported that proinflammatory cytokine expression was induced differently in different tissues. In tracheas, at an early infection stage (1–3 dpi), expression of IL-1β, IL-10R2, IL-6, and LITAF was elicited after inoculation with either respiratory or nephropathogenic IBV strains [61]. The expression of IL-1β was initially downregulated (12 hpi) and sharply increased as the IBV infection progressed in tracheas, when chickens were inoculated with the respiratory Conn IBV strain [26]. Furthermore, expression of IL-6 was upregulated by p38 phosphorylation during IBV infection [78].

In kidneys, regulation of these cytokines was not significantly affected following infection with respiratory IS/885/00-like (885), M41, and nephropathogenic QX-like IBV strains [79]. Chickens infected with nephropathogenic IBV strain of KIIa genotype presented upregulated mRNA levels of IL-6 and IL-1β at 1 dpi in tracheas and kidneys, whereas chickens infected with respiratory IBV strain of ChVI genotype showed comparatively mild upregulated mRNA expression of these cytokines [80]. In addition, in the splenic immune system, the expression levels of IL-7 and IL-18 were significantly upregulated at 1 dpi after respiratory IBV infection [62].

Chemokines orchestrate migration of cells during immune surveillance. Mass IBV strain stimulated gene expression of CXCR4, CCR6, chemokine-like receptor 1/CHEMR23, and Matrix metalloproteinase (MMPs) from an early phase of viral infection (1 dpi) in tracheas [58]. These chemokines may play a role in migration of activated T cells, which may further contribute to elimination of the virus.

**Figure 1:**
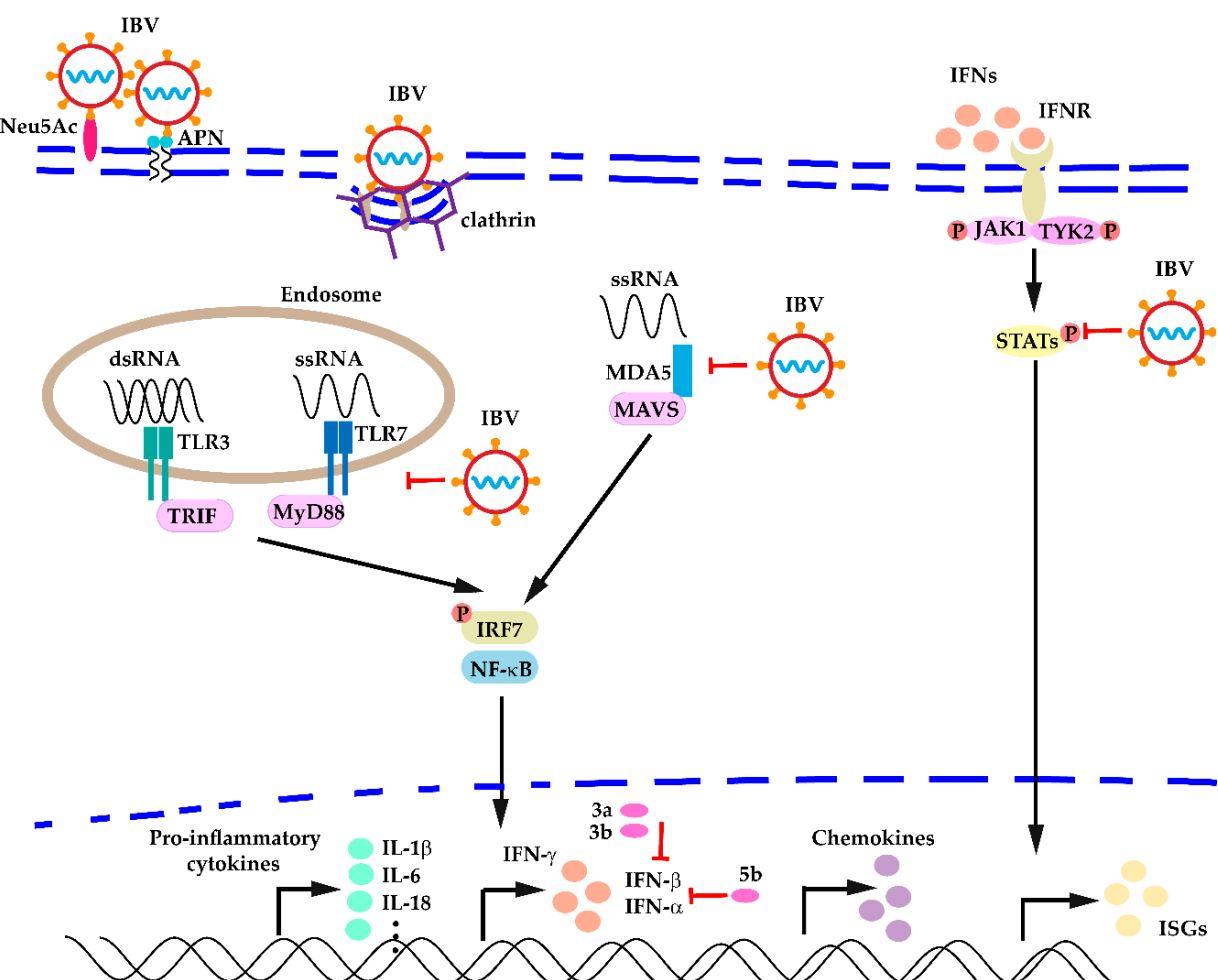
A general description of host innate immune responses against IBV infection.

Based on these observations, after IBV infection occurs, the innate immunity is activated, resulting in recruiting of innate cells at the infection sites and upregulation of different PPRs, cytokines, and chemokines, etc. However, downregulation of PPRs (TLR7), IFNs (IFN-β, IFN-γ), and other cytokines (IL-1β) was still observed at a very early stage of infection (12 hpi) by respiratory IBV strains, suggesting inhibition of the innate immunity is important to establish successful IBV infections, which may reflect common strategies the coronaviruses might take to avoid detection by the host innate immunity. Because most studies were carried out using respiratory IBV strains, it would be important to gain more information about the antiviral host responses using the nephropathogenic IBV strains, which may help to develop vaccination strategies and other intervention programs. A general description of the innate immune responses against IBV infection is shown in Figure 1.

## 5. Apoptosis Triggered by IBV Infection

Apoptosis is one of the primary mechanisms that animals use to combat viral infections. It can also facilitate virus spread at a later stage of infection [81]. There have been reports about IBV-induced apoptosis both in vivo [55] and in vitro [28,82]. 

It is suggested that the IBV ORF1b region is responsible for triggering apoptosis [83]. In mammalian cells, the Bcl 2 family of proteins, including proapoptotic (Bax and Bak) and anti-apoptotic (Mcl 1, Bcl 2, and Bcl XL) proteins, modulated IBV-induced apoptosis at an early stage of IBV infection [55]. In IBV M41-infected HD11 and PBMCs-Mφ cells, a decreased expression of Bcl-2 accompanied by increased expression of Bcl-2-associated X (Bax) suggests viral replication provokes apoptosis at 48 hpi [28]. At a late stage of infection, apoptosis was demonstrated to facilitate IBV replication. Consider IBV Beaudette-infected DF-1 cells for instance [84]. In these cells, the mitogen-activated protein kinase/extracellular signal-regulated protein kinase (MAPK/ERK) pathway was activated; this pathway is negatively regulated by phosphatase DUSP6 [84]. Furthermore, unfolded-protein response (UPR) sensor IRE1α-XBP1 pathway was also activated at late stages of IBV infection [85]. 

## 6. Perspectives in IBV Control

Since first being documented in the United States in 1931, IBV has become endemic throughout the poultry industry [10]. It has been suggested that other avian species might play a role in the spread of IBV worldwide [86]. For instance, a partial nucleotide sequence of coronavirus isolated from parrots (*E. roratus*) showed 100% homology with the IBV GI-13 lineage [87]. Whether wild birds and these avian coronaviruses contribute to the spread of IBV requires further evidence. 

Alongside research on vaccination and prevention measures, in recent years more attention has been focused on understanding the early immune responses after IBV infection, as this would expand our knowledge of the pathology of the virus, which in turn could benefit the development of prevention and control strategies. Innate immunity contributes to a network by utilizing PPRs to detect conserved PAMPs, where different components such as IFNs and proinflammatory cytokines play essential roles in antiviral activity. Several reviews regarding chicken immune responses to IBV infection are recommended for a comprehensive understanding of the virus-host immunity interaction [55].

Given the vast diversity of IBV strains, innate immune responses evoked by IBV infection vary in a strain-dependent and time-dependent manner. Still, early intervention and activation of the innate immunity is essential for control of the disease. To evoke an early innate immune response, agonists of PRRs and IFNs have drawn more attention in novel vaccine design. In addition, population diversity of the virus also contributes to the enhancement of host immunity, as a more diverse viral population in the vaccine induced stronger innate immune responses [88]. Therefore, for a more comprehensive understanding of the IBV-host innate immunity interaction and future development of prevention and control strategies, IBV population structure, the diversity of viral genome, and culture system, as well as the condition of the host animals should be taken into consideration.

Though the information on IBV-host innate immunity interaction is still limited due to lack of experimental measures in chicken, it is well-recognized that the innate immunity contributes not only to prevention strategy development, but also to the pathogenicity of the virus. For effective control of the virus, early enhancement of the host innate immunity is critical. Furthermore, because the chicken innate immunity acts in a strain-dependent and time-dependent manner after IBV infection, early diagnosis of the IBV strain is also important for better control of the virus. Further investigation is required to explore differences in immune response triggered by different IBV strains with differing genotypes and pathogenicity.

## Data Availability

Not applicable.

## References

[1] Cavanagh D., Elus M.M., Cook J.K. (1997). Relationship between sequence variation in the S1 spike protein of infectious bronchitis virus and the extent of cross-protection in vivo. Avian Pathol..

[2] Cavanagh D. (2007). Coronavirus avian infectious bronchitis virus. Vet. Res..

[3] Ganapathy K., Wilkins M., Forrester A., Lemiere S., Cserep T., McMullin P., Jones R.C. (2012). QX-like infectious bronchitis virus isolated from cases of proventriculitis in commercial broilers in England. Vet. Rec..

[4] Ambali A.G., Jones R.C. (1990). Early pathogenesis in chicks of infection with an enterotropic strain of infectious bronchitis virus. Avian Dis..

[5] Pantin-Jackwood M.J., Brown T.P., Huff G.R. (2005). Reproduction of proventriculitis in commercial and specific-pathogen-free broiler chickens. Avian Dis..

[6] Raj G.D., Jones R.C. (1997). Infectious bronchitis virus: Immunopathogenesis of infection in the chicken. Avian Pathol..

[7] Matthijs M.G., van Eck J.H., Landman W.J., Stegeman J.A. (2003). Ability of Massachusetts-type infectious bronchitis virus to increase colibacillosis susceptibility in commercial broilers: A comparison between vaccine and virulent field virus. Avian Pathol..

[8] Matthijs M.G., Ariaans M.P., Dwars R.M., van Eck J.H., Bouma A., Stegeman A., Vervelde L. (2009). Course of infection and immune responses in the respiratory tract of IBV infected broilers after superinfection with *E. coli*. Vet. Immunol. Immunopathol..

[9] De Wit J., Cook J. (2019). Spotlight on avian pathology: Infectious bronchitis virus. Avian Pathol..

[10] Cook J.K., Jackwood M., Jones R.C. (2012). The long view: 40 years of infectious bronchitis research. Avian Pathol..

[11] Lim Y.X., Ng Y.L., Tam J.P., Liu D.X. (2016). Human Coronaviruses: A Review of Virus-Host Interactions. Diseases.

[12] Meeusen E.N., Walker J., Peters A., Pastoret P.P., Jungersen G. (2007). Current status of veterinary vaccines. Clin. Microbiol. Rev..

[13] Silva A., Hauck R., Kern C., Wang Y., Zhou H., Gallardo R.A. (2019). Effects of Chicken MHC Haplotype on Resistance to Distantly Related Infectious Bronchitis Viruses. Avian Dis..

[14] Da Silva A.P., Schat K.A., Gallardo R.A. (2020). Cytokine Responses in Tracheas from Major Histocompatibility Complex Congenic Chicken Lines with Distinct Susceptibilities to Infectious Bronchitis Virus. Avian Dis..

[15] Asif M., Lowenthal J.W., Ford M.E., Schat K.A., Kimpton W.G., Bean A.G. (2007). Interleukin-6 expression after infectious bronchitis virus infection in chickens. Viral Immunol..

[16] Wickramasinghe I.N., de Vries R.P., Gröne A., de Haan C.A., Verheije M.H. (2011). Binding of avian coronavirus spike proteins to host factors reflects virus tropism and pathogenicity. J. Virol..

[17] Sun X., Li L., Pan L., Wang Z., Chen H., Shao C., Yu J., Ren Y., Wang X., Huang X. (2021). Infectious bronchitis virus: Identification of Gallus gallus APN high-affinity ligands with antiviral effects. Antivir. Res..

[18] Zhu P., Lv C., Fang C., Peng X., Sheng H., Xiao P., Kumar Ojha N., Yan Y., Liao M., Zhou J. (2020). Heat Shock Protein Member 8 Is an Attachment Factor for Infectious Bronchitis Virus. Front. Microbiol..

[19] Wang H., Yuan X., Sun Y., Mao X., Meng C., Tan L., Song C., Qiu X., Ding C., Liao Y. (2019). Infectious bronchitis virus entry mainly depends on clathrin mediated endocytosis and requires classical endosomal/lysosomal system. Virology.

[20] Van Ginkel F.W., Padgett J., Martinez-Romero G., Miller M.S., Joiner K.S., Gulley S.L. (2015). Age-dependent immune responses and immune protection after avian coronavirus vaccination. Vaccine.

[21] Genovese K.J., He H., Swaggerty C.L., Kogut M.H. (2013). The avian heterophil. Dev. Comp. Immunol..

[22] Sekelova Z., Stepanova H., Polansky O., Varmuzova K., Faldynova M., Fedr R., Rychlik I., Vlasatikova L. (2017). Differential protein expression in chicken macrophages and heterophils in vivo following infection with Salmonella Enteritidis. Vet. Res..

[23] Fulton R.M., Reed W.M., Thacker H.L. (1993). Cellular response of the respiratory tract of chickens to infection with Massachusetts 41 and Australian T infectious bronchitis viruses. Avian Dis..

[24] Watters T.M., Kenny E.F., O’Neill L.A. (2007). Structure, function and regulation of the Toll/IL-1 receptor adaptor proteins. Immunol. Cell Biol..

[25] Dar A., Potter A., Tikoo S., Gerdts V., Lai K., Babiuk L.A., Mutwiri G. (2009). CpG oligodeoxynucleotides activate innate immune response that suppresses infectious bronchitis virus replication in chicken embryos. Avian Dis..

[26] Kameka A.M., Haddadi S., Kim D.S., Cork S.C., Abdul-Careem M.F. (2014). Induction of innate immune response following infectious bronchitis corona virus infection in the respiratory tract of chickens. Virology.

[27] Amarasinghe A., Abdul-Cader M.S., Almatrouk Z., van der Meer F., Cork S.C., Gomis S., Abdul-Careem M.F. (2018). Induction of innate host responses characterized by production of interleukin (IL)-1β and recruitment of macrophages to the respiratory tract of chickens following infection with infectious bronchitis virus (IBV). Vet. Microbiol..

[28] Sun X., Wang Z., Shao C., Yu J., Liu H., Chen H., Li L., Wang X., Ren Y., Huang X. (2021). Analysis of chicken macrophage functions and gene expressions following infectious bronchitis virus M41 infection. Vet. Res..

[29] Li H., Cui P., Fu X., Zhang L., Yan W., Zhai Y., Lei C., Wang H., Yang X. (2021). Identification and analysis of long non-coding RNAs and mRNAs in chicken macrophages infected with avian infectious bronchitis coronavirus. BMC Genom..

[30] Yang X., Gao W., Liu H., Li J., Chen D., Yuan F., Zhang Z., Wang H. (2017). MicroRNA transcriptome analysis in chicken kidneys in response to differing virulent infectious bronchitis virus infections. Arch. Virol..

[31] Li H., Li J., Zhai Y., Zhang L., Cui P., Feng L., Yan W., Fu X., Tian Y., Wang H. (2020). Gga-miR-30d regulates infectious bronchitis virus infection by targeting USP47 in HD11 cells. Microb. Pathog..

[32] Liu H., Yang X., Zhang Z.K., Zou W.C., Wang H.N. (2018). miR-146a-5p promotes replication of infectious bronchitis virus by targeting IRAK2 and TNFRSF18. Microb. Pathog..

[33] Qureshi M.A., Miller L., Lillehoj H.S., Ficken M.D. (1990). Establishment and characterization of a chicken mononuclear cell line. Vet. Immunol. Immunopathol..

[34] Ziegenhain C., Vieth B., Parekh S., Reinius B., Guillaumet-Adkins A., Smets M., Leonhardt H., Heyn H., Hellmann I., Enard W. (2017). Comparative Analysis of Single-Cell RNA Sequencing Methods. Mol. Cell.

[35] Vervelde L., Matthijs M.G., van Haarlem D.A., de Wit J.J., Jansen C.A. (2013). Rapid NK-cell activation in chicken after infection with infectious bronchitis virus M41. Vet. Immunol. Immunopathol..

[36] Göbel T.W., Kaspers B., Stangassinger M. (2001). NK and T cells constitute two major, functionally distinct intestinal epithelial lymphocyte subsets in the chicken. Int. Immunol..

[37] Hoffmann J., Akira S. (2013). Innate immunity. Curr. Opin. Immunol..

[38] Chan Y.K., Gack M.U. (2016). Viral evasion of intracellular DNA and RNA sensing. Nature reviews. Microbiology.

[39] Kang J.Y., Lee J.O. (2011). Structural biology of the Toll-like receptor family. Annu. Rev. Biochem..

[40] St Paul M., Brisbin J.T., Abdul-Careem M.F., Sharif S. (2013). Immunostimulatory properties of Toll-like receptor ligands in chickens. Vet. Immunol. Immunopathol..

[41] Higgs R., Cormican P., Cahalane S., Allan B., Lloyd A.T., Meade K., James T., Lynn D.J., Babiuk L.A., O’farrelly C. (2006). Induction of a novel chicken Toll-like receptor following Salmonella enterica serovar Typhimurium infection. Infect. Immun..

[42] Brownlie R., Zhu J., Allan B., Mutwiri G.K., Babiuk L.A., Potter A., Griebel P. (2009). Chicken TLR21 acts as a functional homologue to mammalian TLR9 in the recognition of CpG oligodeoxynucleotides. Mol. Immunol..

[43] Abdel-Mageed A.M., Isobe N., Yoshimura Y. (2014). Effects of different TLR ligands on the expression of proinflammatory cytokines and avian β-defensins in the uterine and vaginal tissues of laying hens. Vet. Immunol. Immunopathol..

[44] Takeda K., Akira S. (2015). Toll-like receptors. Curr. Protoc. Immunol..

[45] Gillespie M., Shamovsky V., D’Eustachio P. (2011). Human and chicken TLR pathways: Manual curation and computer-based orthology analysis. Mamm. Genome Off. J. Int. Mamm. Genome Soc..

[46] Karpala A.J., Lowenthal J.W., Bean A.G. (2012). Identifying innate immune pathways of the chicken may lead to new antiviral therapies. Vet. Immunol. Immunopathol..

[47] Nawab A., An L., Wu J., Li G., Liu W., Zhao Y., Wu Q., Xiao M. (2019). Chicken toll-like receptors and their significance in immune response and disease resistance. Int. Rev. Immunol..

[48] Matsumiya T., Stafforini D.M. (2010). Function and regulation of retinoic acid-inducible gene-I. Crit. Rev. Immunol..

[49] Barber M.R., Aldridge J.R., Webster R.G., Magor K.E. (2010). Association of RIG-I with innate immunity of ducks to influenza. Proc. Natl. Acad. Sci. USA.

[50] Karpala A.J., Stewart C., McKay J., Lowenthal J.W., Bean A.G. (2011). Characterization of chicken Mda5 activity: Regulation of IFN-β in the absence of RIG-I functionality. J. Immunol..

[51] Liniger M., Summerfield A., Zimmer G., McCullough K.C., Ruggli N. (2012). Chicken cells sense influenza A virus infection through MDA5 and CARDIF signaling involving LGP2. J. Virol..

[52] Stewart C.R., Bagnaud-Baule A., Karpala A.J., Lowther S., Mohr P.G., Wise T.G., Lowenthal J.W., Bean A.G. (2012). Toll-like receptor 7 ligands inhibit influenza A infection in chickens. J. Interferon Cytokine Res..

[53] Tao Z.Y., Zhu C.H., Shi Z.H., Song C., Xu W.J., Song W.T., Zou J.M., Qin A.J. (2015). Molecular characterization, expression, and functional analysis of NOD1 in Qingyuan partridge chicken. Genet. Mol. Res..

[54] Chen S., Cheng A., Wang M. (2013). Innate sensing of viruses by pattern recognition receptors in birds. Vet. Res..

[55] Chhabra R., Kuchipudi S.V., Chantrey J., Ganapathy K. (2016). Pathogenicity and tissue tropism of infectious bronchitis virus is associated with elevated apoptosis and innate immune responses. Virology.

[56] Kint J., Fernandez-Gutierrez M., Maier H.J., Britton P., Langereis M.A., Koumans J., Wiegertjes G.F., Forlenza M. (2015). Activation of the chicken type I interferon response by infectious bronchitis coronavirus. J. Virol..

[57] Yu L., Zhang X., Wu T., Su J., Wang Y., Wang Y., Ruan B., Niu X., Wu Y. (2017). Avian infectious bronchitis virus disrupts the melanoma differentiation associated gene 5 (MDA5) signaling pathway by cleavage of the adaptor protein MAVS. BMC Vet. Res..

[58] Guo X., Rosa A.J., Chen D.G., Wang X. (2008). Molecular mechanisms of primary and secondary mucosal immunity using avian infectious bronchitis virus as a model system. Vet. Immunol. Immunopathol..

[59] Wang X., Rosa A.J., Oliverira H.N., Rosa G.J., Guo X., Travnicek M., Girshick T. (2006). Transcriptome of local innate and adaptive immunity during early phase of infectious bronchitis viral infection. Viral Immunol..

[60] Okino C.H., Mores M.A., Trevisol I.M., Coldebella A., Montassier H.J., Brentano L. (2017). Early immune responses and development of pathogenesis of avian infectious bronchitis viruses with different virulence profiles. PLoS ONE.

[61] Smith J., Sadeyen J.R., Cavanagh D., Kaiser P., Burt D.W. (2015). The early immune response to infection of chickens with Infectious Bronchitis Virus (IBV) in susceptible and resistant birds. BMC Vet. Res..

[62] Khan S., Roberts J., Wu S.B. (2020). Regulation of Immunity-Related Genes by Infectious Bronchitis Virus Challenge in Spleen of Laying Chickens. Viral Immunol..

[63] Cong F., Liu X., Han Z., Shao Y., Kong X., Liu S. (2013). Transcriptome analysis of chicken kidney tissues following coronavirus avian infectious bronchitis virus infection. BMC Genom..

[64] Zhu J., Xu S., Li X., Wang J., Jiang Y., Hu W., Ruan W. (2020). Infectious bronchitis virus inhibits activation of the TLR7 pathway, but not the TLR3 pathway. Arch. Virol..

[65] Matoo J.J., Bashir K., Kumar A., Krishnaswamy N., Dey S., Chellappa M.M., Ramakrishnan S. (2018). Resiquimod enhances mucosal and systemic immunity against avian infectious bronchitis virus vaccine in the chicken. Microb. Pathog..

[66] Gürtler C., Bowie A.G. (2013). Innate immune detection of microbial nucleic acids. Trends Microbiol..

[67] Schneider W.M., Chevillotte M.D., Rice C.M. (2014). Interferon-stimulated genes: A complex web of host defenses. Annu. Rev. Immunol..

[68] Huang B., Qi Z.T., Xu Z., Nie P. (2010). Global characterization of interferon regulatory factor (IRF) genes in vertebrates: Glimpse of the diversification in evolution. BMC Immunol..

[69] Liu A.L., Li Y.F., Qi W., Ma X.L., Yu K.X., Huang B., Liao M., Li F., Pan J., Song M.X. (2015). Comparative analysis of selected innate immune-related genes following infection of immortal DF-1 cells with highly pathogenic (H5N1) and low pathogenic (H9N2) avian influenza viruses. Virus Genes.

[70] Liu Y., Cheng Y., Shan W., Ma J., Wang H., Sun J., Yan Y. (2018). Chicken interferon regulatory factor 1 (IRF1) involved in antiviral innate immunity via regulating IFN-β production. Dev. Comp. Immunol..

[71] Mordstein M., Neugebauer E., Ditt V., Jessen B., Rieger T., Falcone V., Sorgeloos F., Ehl S., Mayer D., Kochs G. (2010). Lambda interferon renders epithelial cells of the respiratory and gastrointestinal tracts resistant to viral infections. J. Virol..

[72] Santhakumar D., Rubbenstroth D., Martinez-Sobrido L., Munir M. (2017). Avian Interferons and Their Antiviral Effectors. Front. Immunol..

[73] Pei J., Sekellick M.J., Marcus P.I., Choi I.S., Collisson E.W. (2001). Chicken interferon type I inhibits infectious bronchitis virus replication and associated respiratory illness. J. Interferon Cytokine Res..

[74] Kint J., Langereis M.A., Maier H.J., Britton P., van Kuppeveld F.J., Koumans J., Wiegertjes G.F., Forlenza M. (2016). Infectious Bronchitis Coronavirus Limits Interferon Production by Inducing a Host Shutoff That Requires Accessory Protein 5b. J. Virol..

[75] Okino C.H., dos Santos I.L., Fernando F.S., Alessi A.C., Wang X., Montassier H.J. (2014). Inflammatory and cell-mediated immune responses in the respiratory tract of chickens to infection with avian infectious bronchitis virus. Viral Immunol..

[76] Barjesteh N., Behboudi S., Brisbin J.T., Villanueva A.I., Nagy E., Sharif S. (2014). TLR ligands induce antiviral responses in chicken macrophages. PLoS ONE.

[77] Dar A., Tikoo S., Potter A., Babiuk L.A., Townsend H., Gerdts V., Mutwiri G. (2014). CpG-ODNs induced changes in cytokine/chemokines genes expression associated with suppression of infectious bronchitis virus replication in chicken lungs. Vet. Immunol. Immunopathol..

[78] Liao Y., Wang X., Huang M., Tam J.P., Liu D.X. (2011). Regulation of the p38 mitogen-activated protein kinase and dual-specificity phosphatase 1 feedback loop modulates the induction of interleukin 6 and 8 in cells infected with coronavirus infectious bronchitis virus. Virology.

[79] Chhabra R., Ball C., Chantrey J., Ganapathy K. (2018). Differential innate immune responses induced by classical and variant infectious bronchitis viruses in specific pathogen free chicks. Dev. Comp. Immunol..

[80] Jang H., Koo B.S., Jeon E.O., Lee H.R., Lee S.M., Mo I.P. (2013). Altered pro-inflammatory cytokine mRNA levels in chickens infected with infectious bronchitis virus. Poult. Sci..

[81] Elmore S. (2007). Apoptosis: A review of programmed cell death. Toxicol. Pathol..

[82] Han X., Tian Y., Guan R., Gao W., Yang X., Zhou L., Wang H. (2017). Infectious Bronchitis Virus Infection Induces Apoptosis during Replication in Chicken Macrophage HD11 Cells. Viruses.

[83] Li F.Q., Tam J.P., Liu D.X. (2007). Cell cycle arrest and apoptosis induced by the coronavirus infectious bronchitis virus in the absence of p53. Virology.

[84] Wang H., Liu D., Sun Y., Meng C., Tan L., Song C., Qiu X., Liu W., Ding C., Ying L. (2021). Upregulation of DUSP6 impairs infectious bronchitis virus replication by negatively regulating ERK pathway and promoting apoptosis. Vet. Res..

[85] Fung T.S., Liao Y., Liu D.X. (2014). The endoplasmic reticulum stress sensor IRE1α protects cells from apoptosis induced by the coronavirus infectious bronchitis virus. J. Virol..

[86] Awad F., Forrester A., Baylis M., Lemiere S., Jones R., Ganapathy K. (2015). Immune responses and interactions following simultaneous application of live Newcastle disease, infectious bronchitis and avian metapneumovirus vaccines in specific-pathogen-free chicks. Res. Vet. Sci..

[87] Suryaman G.K., Soejoedono R.D., Setiyono A., Poetri O.N., Handharyani E. (2019). Isolation and characterization of avian coronavirus from healthy Eclectus parrots (Eclectus roratus) from Indonesia. Vet. World.

[88] Zegpi R.A., Joiner K.S., van Santen V.L., Toro H. (2020). Infectious Bronchitis Virus Population Structure Defines Immune Response and Protection. Avian Dis..

